# Association between routine blood count-derived inflammatory markers and left ventricular hypertrophy in diabetic kidney disease patients: a cross-sectional study

**DOI:** 10.3389/fcdhc.2026.1790675

**Published:** 2026-06-17

**Authors:** Rui Wu, Gaiqi Yao

**Affiliations:** 1 Department of General Practice, Peking University Third Hospital, Beijing, China; 2 Department of Intensive Care Unit, Peking University Third Hospital, Beijing, China

**Keywords:** diabetic kidney disease, left ventricular hypertrophy, monocyte-to-lymphocyte ratio, neutrophil-to-lymphocyte ratio, platelet-to-lymphocyte ratio, systemic immune inflammation index

## Abstract

**Background:**

Adverse cardiovascular events are one of the most serious complications of diabetic kidney disease (DKD), and left ventricular hypertrophy (LVH) is a reliable predictor. The low-grade inflammatory state in patients with DKD further aggravates the damage to the kidneys and cardiovascular system. This study aims to explore the relationship between routine blood count-derived inflammatory markers in the peripheral blood of DKD patients and LVH.

**Methods:**

In this cross-sectional study, 419 patients with DKD G2 ~ G4, who received treatment at the Peking University Third Hospital from January 2020 to September 2025 were enrolled. Based on the echocardiographic findings, the participants were categorized into two groups: those with LVH and those without. Logistic regression was employed to examine the factors associated with LVH, as well as the interactions among neutrophil, the systemic immune-inflammation index (SII), and the stages of DKD. Additionally, receiver operating characteristic (ROC) curves and the optimal cut-off values of the SII and neutrophil were generated.

**Results:**

Among the 419 participants included in the study, there were 239 males and 180 females, with a baseline age of 66 years. 106 individuals were categorized in the LVH group. Multivariate logistic regression analysis revealed that neutrophil (*OR* = 1.31, *P* = 0.011), SII (*OR* = 1.01, *P* = 0.011), uric acid (*OR* = 1.01, *P<*0.01), and urea (*OR* = 1.02, *P* = 0.002) are positive correlation factors for LVH. In contrast, male gender (*OR* = 0.33, *P<*0.01) and calcium (*OR* = 0.07, *P* = 0.001) serve as negative correlation factors for LVH. Furthermore, DKD stage did not significantly modify the effects of neutrophil (interaction *P* = 0.09) and SII (interaction *P* = 0.22). The area under the curve (AUC) for basic variables, which include gender, calcium, uric acid, and urea, when combined with neutrophil and SII, was 0.816. This AUC, along with that of basic variables combined with SII (0.810), exceeded the AUC of basic variables alone (0.782), with the difference reaching statistical significance (*P* < 0.05). The optimal cut-off value for neutrophil is 4.14×10^9^/L, and the optimal cutoff value for SII is 491.78×10^9^/L.

**Conclusion:**

In DKD G2 ~ G4 patients, neutrophil, SII, uric acid and urea sever as independent positive correlation factors for LVH. In contrast, male gender and calcium serves as negative correlation factors.

## 1 Introduction

Diabetic Kidney Disease (DKD) represents a significant microvascular complication of diabetes, characterized by progressive kidney damage resulting from persistent proteinuria and a diminished glomerular filtration rate. It has emerged as the leading cause of end-stage renal disease, contributing to a substantial global burden of morbidity and mortality (1). In individuals with DKD, adverse cardiovascular events rank among the most serious complications. Left ventricular hypertrophy (LVH), a primary indicator of cardiac structural remodeling, has been established as a robust predictor of adverse cardiovascular outcomes, markedly elevating the risk of heart failure and mortality (2, 3). Previous studies have demonstrated that chronic kidney disease is marked by a persistent low-grade inflammatory state, a condition that also applies to DKD. This inflammatory state is intricately linked to the activation of immune cells, including macrophages and T lymphocytes, as well as the release of pro-inflammatory cytokines such as Tumor Necrosis Factor-α (TNF-α) and Interleukin-6 (IL-6). Additionally, it contributes to the exacerbation of oxidative stress, which further intensifies renal and cardiovascular damage by promoting endothelial dysfunction and fibrosis pathways (4, 5).

In recent years, composite inflammatory indicators derived from routine complete blood counts, including the Neutrophil-to-Lymphocyte Ratio (NLR), Monocyte-to-Lymphocyte Ratio (MLR), Platelet-to-Lymphocyte Ratio (PLR), and the Systemic Immune Inflammation Index (SII), have garnered significant attention (6–8). Compared with the individual counts of white blood cells or neutrophils, the composite index incorporates the three primary immune and coagulation pathways involving neutrophils, lymphocytes, and platelets. This index offers a more comprehensive and stable reflection of the imbalance between the body’s pro-inflammatory and immune regulatory states. While these indicators have demonstrated prognostic value regarding the progression of renal function, all-cause mortality, and cardiovascular mortality (9–11). However, the relationship between those inflammatory markers and LVH in patients with DKD remains unclear, and there is a lack of observational data. Consequently, this study aimed to investigate the association between routine blood count-derived inflammatory markers-specifically, NLR, MLR, PLR, and SII-and LVH of patients with DKD. This exploration seeks to provide new insights for cardiovascular risk stratification and early intervention.

## 2 Material and methods

### 2.1 Research participants

#### 2.1.1 Research Participants

This cross-sectional study involved 419 patients diagnosed with DKD G2 ~ G4, who received treatment in the outpatient and inpatient departments of Peking University Third Hospital from January 2020 to September 2025.

#### 2.1.2 Inclusion Criteria

(1) Participants must meet the diagnostic criteria for DKD G2 ~ G4 as outlined in the ADA “Standards of Medical Care in Diabetes-2025” (1). (2) Participants must be at least 20 years of age.

#### 2.1.3 Exclusion criteria

(1) Individuals with incomplete clinical data. (2) Patients experiencing acute infections, chronic infectious diseases, acute cardiovascular or cerebrovascular conditions, trauma, or surgery within the preceding 3 months. (3) Patients diagnosed with autoimmune diseases, malignant tumors, severe liver diseases, hematological disorders, or those who have received immunosuppressive therapy in the past 3 months. (4) Individuals who are pregnant, have mental illnesses, or are unable or unwilling to participate in this survey.

## 3 Methods

### 3.1 Data collection

The data collected encompasses gender, age, ethnicity, comorbidities (such as diabetic retinopathy (DR), hypertension, and cardiovascular disease (CVD)), and current medications, which include renin-angiotensin-aldosterone system (RAAS) inhibitors (angiotensin-converting enzyme inhibitors, angiotensin receptor blockers, angiotensin receptor neprilysin inhibitors, and aldosterone receptor blockers) as well as sodium-glucose transporter 2 inhibitors (SGLT2i). Additionally, height, weight, and blood pressure were recorded. A complete blood count was obtained for all participants, including white blood cell (WBC) and its classification (neutrophil, lymphocyte, and monocyte), red blood cell (RBC), hemoglobin, and platelet. In addition, it included the albumin, uric acid (UA), urea, vitamin D (VitD), intact parathyroid hormone (iPTH) of all participants, as well as the measurement results of transthoracic echocardiography.

### 3.2 Indicator measurement and definition

Height and weight were assessed using identical measuring instruments in the hospital setting. Body mass index (BMI) = weight (kg)/height² (m²). According to the standards established by the Chinese Working Group on Obesity and the current Chinese Adult Overweight and Obesity Prevention and Control Guidelines, BMI is categorized as follows: underweight (<18.5 kg/m²), normal (18.5 ~ 23.9 kg/m²), overweight (24.0 ~ 27.9 kg/m²), and obese (≥28.0 kg/m²) (12). Body surface area (BSA) = (0.0061 × height (cm) + 0.0128 × weight (kg) - 0.1529). Blood pressure was measured with a uniformly calibrated upper-arm electronic sphygmomanometer. Participants were instructed to rest for at least 5 minutes prior to measurement. Blood pressure was recorded from the right upper arm while the patient was seated. The measurement was repeated three times, and the average value was taken. NLR = the neutrophil divided by the lymphocyte; MLR = the monocyte divided by the lymphocyte; PLR = the platelet divided by the lymphocyte; and SII = the platelet multiplied by the neutrophil divided by the lymphocyte. The estimated glomerular filtration rate (eGFR) was determined using the CKD-EPI formula (13). Following the KDIGO guidelines, patients were categorized into stage G2 (eGFR 60 to 89 mL/min/1.73m²), stage G3a (eGFR 45 to 59 mL/min/1.73m²), stage G3b (eGFR 30 to 44 mL/min/1.73m²), and stage G4 (eGFR 15 to 29 mL/min/1.73m²) (14).

### 3.3 Measurement method of transthoracic echocardiography

Echocardiographic data were obtained from the Department of Cardiology and measured by two senior cardiac sonographers. The interventricular septal thickness, left ventricular posterior wall thickness, and left ventricular end-diastolic diameter were assessed in the parasternal left ventricular long-axis view. Left ventricular mass = 0.8 × 1.04 × [(left ventricular end-diastolic diameter + left ventricular posterior wall thickness + interventricular septal thickness)³ - left ventricular end-diastolic diameter³] + 0.6. The left ventricular mass index = left ventricular mass/BSA (15).

### 3.4 Diagnostic criteria for LVH

Diagnostic criteria for LVH include transthoracic wall echocardiography revealing a left ventricular mass index of ≥115 g/m² in male and ≥95 g/m² in female (16). Additionally, increased end-diastolic myocardial thickness in any region of the left ventricle is defined as >12 mm in male and >11 mm in female (17).

### 3.5 Sample size calculation

Based on the empirical criterion that each independent variable in logistic regression necessitates a minimum of 10 outcome events (EPV ≥ 10), this study anticipates an LVH incidence of 25% and plans to incorporate 10 independent variables. Consequently, the estimated required sample size is at least 400 cases. Ultimately, 419 cases were included.

### 3.6 Statistical methods

Statistical analysis was conducted using SPSS version 27.0. The Shapiro-Wilk test assessed whether the measurement data followed a normal distribution. The measurement data that follow a normal distribution were expressed as x¯ ± S, and the comparison between groups was conducted using the t-test; for the measurement data that did not follow a normal distribution, they were represented as M (Q1, Q3), and the comparison between groups was performed using non-parametric tests. The Categorical data were expressed as rates or proportions (%), and the comparison between groups was conducted using the χ^2^ test. The presence or absence of LVH was designated as the dependent variable, with “1” indicating the presence of LVH and “0” indicating its absence. Univariate logistic regression analysis was conducted using the independent variables identified in the single-factor analysis. Subsequently, multivariate logistic regression analysis (forward: LR) was performed, incorporating age, gender, ethnicity, DKD stages, the presence of hypertension, CVD, and other variables that exhibited significant differences in the univariate logistic regression analysis as covariates. The model was enhanced by incorporating interaction terms to examine the relationship between neutrophil, SII, and DKD stage. Additionally, collinearity among the independent variables was assessed. The operating receiver characteristic (ROC) curve and integrated discrimination improvement (IDI) were employed to evaluate the predictive performance of basic variables, which included gender, calcium, UA, and urea. Additionally, we assessed the performance of models that combined basic variables with neutrophil, basic variables with SII, and basic variables with both neutrophil and SII in patients with DKD and LVH. The area under the curve (AUC) was compared using paired sample area differences under the ROC curve to determine statistical significance. The Youden index maximization method was employed to determine the optimal cut-off values for neutrophil and SII in predicting LVH in patients with DKD. *P<*0.05 was considered indicative of a statistically significant difference.

## 4 Results

### 4.1 LVH and baseline characteristics of participants

Among the 419 participants included in the cross-sectional study, 239 were male and 180 were female, with an average age of 66 years. Of these participants, 106 exhibited LVH, and the proportion of women in the LVH group was significantly higher than that in the non-LVH group (*P<*0.05), as shown in the **Table 1**.

**Table 1:** LVH and baseline characteristics of 419 participants.

Variables	Total (n = 419)	No-LVH (n = 313)	LVH (n = 106)	Z/χ^2^	*P*
age, M (Q_1_, Q_3_)	66.0 (56.0, 75.0)	66.0 (56.0, 75.0)	66.0 (55.3, 75.0)	-0.16	0.872
Gender [n (%)]				12.32	<0.01
Female	180 (43.0)	119 (38.0)	61 (57.6)		
Male	239 (57.0)	194 (62.0)	45 (42.4)		
Ethnicity [n (%)]				0.10	0.751
Non-han ethnicity	47 (11.2)	36 (11.5)	11 (10.4)		
Han ethnicity	372 (88.8)	277 (88.5)	95 (89.6)		
Height (cm), M (Q_1_, Q_3_)	166.0 (160.0, 172.0)	167.0 (160.0, 172.0)	165.0 (159.0, 170.0)	-1.45	0.147
Weight (kg), M (Q_1_, Q_3_)	71.0 (62.0, 79.0)	72.0 (63.0, 79.5)	68.3 (60.0, 78.0)	-1.54	0.122
BMI (kg/m^2^), M (Q_1_, Q_3_)	25.7 (23.3, 28.0)	25.8 (23.3, 27.9)	25.0 (23.2, 28.3)	-0.57	0.567
BMI (kg/m^2^) [n (%)]				2.24	0.524
BMI<18.5	12 (2.9)	8 (2.6)	4 (3.8)		
18.5≤BMI ≤ 23.9	124 (29.6)	90 (28.8)	34 (32.1)		
24≤BMI ≤ 27.9	179 (42.7)	140 (44.7)	39 (36.8)		
BMI≥28	104 (24.8)	75 (24.0)	29 (27.4)		
Systolic blood pressure (mmHg), M (Q_1_, Q_3_)	137.0 (125.0, 153.0)	137.0 (125.0, 151.0)	138.5 (124.0, 158.8)	-1.28	0.202
Diastolic blood pressure (mmHg), M (Q_1_, Q_3_)	78.0 (69.0, 87.0)	78.0 (69.0, 87.0)	77.0 (68.3, 84.0)	-0.69	0.487
Hypertention [n (%)]				0.30	0.585
Without hypertention	95 (22.7)	73 (23.3)	22 (20.8)		
With hypertention	324 (77.3)	240 (76.7)	84 (79.3)		
CVD [n (%)]				0.23	0.634
Without CVD	265 (63.3)	200 (63.9)	65 (61.3)		
With CVD	154 (36.8)	113 (36.1)	41 (38.7)		
DR [n (%)]				0.73	0.394
Without DR	16 (3.8)	10 (3.2)	6 (5.7)		
With DR	403 (96.2)	303 (96.8)	100 (94.3)		
DKD stage [n (%)]				6.68	0.083
G2	127 (30.3)	91 (29.1)	36 (34.0)		
G3a	123 (29.4)	98 (31.3)	25 (23.6)		
G3b	104 (24.8)	82 (26.2)	22 (20.8)		
G4	65 (15.5)	42 (13.4)	23 (21.7)		

Z, Mann-Whitney test; χ², Chi-square test; M, Median; Q₁, 1st Quartile; Q₃, 3st Quartile; Bold values indicate *P*<0.05. LVH, Left ventricular hypertrophy; BMI, Body mass index; CVD, Cardiovascular disease; DR, Diabetic retinopathy.

### 4.2 Results of single-factor analysis of LVH in the participants

The neutrophil, NLR, PLR, SII, UA, and urea levels in the LVH group were significantly elevated compared to those in the non-LVH group (all *P<*0.05). Conversely, calcium levels in the LVH group were significantly lower than those in the non-LVH group (*P<*0.05). No significant differences were observed between the two groups regarding the use of RAAS drugs, SGLT2i, WBC, RBC, hemoglobin, platelet, lymphocyte, monocyte, MLR, albumin, phosphorus, vitD, and iPTH (all *P*>0.05). These findings are summarized in the **Table 2**.

**Table 2:** Results of single-factor analysis of LVH in the participants.

Variables	Total (n = 419)	No-LVH (n = 313)	LVH (n = 106)	Z/χ^2^	*P*
RAAS inhibitors [n (%)]				0.33	0.567
Without RAAS inhibitors	160 (38.2)	122 (39.0)	38 (35.9)		
With RAAS inhibitors	259 (61.8)	191 (61.0)	68 (64.2)		
SGLT2i [n (%)]				3.04	0.081
Without SGLT2i	278 (66.4)	215 (68.7)	63 (59.4)		
With SGLT2i	141 (33.7)	98 (31.3)	43 (40.6)		
WBC (×10^9^/L), M (Q_1_, Q_3_)	6.73 (5.52, 8.17)	6.73 (5.53, 8.04)	6.74 (5.51, 8.46)	-0.47	0.641
RBC (×10^9^/L), M (Q_1_, Q_3_)	4.27 (3.84, 4.70)	4.27 (3.81, 4.72)	4.27 (3.94, 4.64)	-0.11	0.916
Hemoglobin (g/L), M (Q_1_, Q_3_)	118.0 (105.5, 132.0)	117.0 (106.0, 132.0)	119.0 (105.3, 130.8)	-0.14	0.892
Platelets (×10^9^/L), M (Q_1_, Q_3_)	235.0 (198.0, 282.0)	231.0 (190.0, 283.0)	237.5 (218.3, 278.3)	-1.79	0.074
Net (×10^9^/L), M (Q_1_, Q_3_)	4.22 (3.48, 5.30)	4.07 (3.35, 5.01)	4.76 (4.05, 5.78)	-4.59	**<0.01**
Lym (×10^9^/L), M (Q_1_, Q_3_)	1.77 (1.35, 2.21)	1.80 (1.37, 2.24)	1.69 (1.32, 2.13)	-0.87	0.386
Mono (×10^9^/L), M (Q_1_, Q_3_)	0.37 (0.28, 0.46)	0.37 (0.29, 0.46)	0.36 (0.28, 0.48)	-0.31	0.759
NLR, M (Q_1_, Q_3_)	2.41 (1.86, 3.28)	2.38 (1.72, 3.22)	2.60 (2.05, 3.71)	-2.03	**0.042**
MLR, M (Q_1_, Q_3_)	0.21 (0.16, 0.30)	0.21 (0.15, 0.29)	0.22 (0.17, 0.31)	-0.62	0.533
PLR, M (Q_1_, Q_3_)	133.6 (101.8, 176.4)	126.8 (97.9, 169.9)	149.3 (125.4, 186.3)	-3.25	**0.001**
SII (×10^9^/L), M (Q_1_, Q_3_)	511.0 (377.9, 725.0)	466.6 (327.4, 661.8)	641.6 (557.8, 874.5)	-6.52	**<0.01**
Albumin (g/L), M (Q_1_, Q_3_)	38.0 (35.0, 42.0)	38.8 (34.0, 42.0)	37.0 (35.0, 40.0)	-0.96	0.338
Calcium (mmol/L), M (Q_1_, Q_3_)	2.27 (2.17, 2.38)	2.29 (2.18, 2.40)	2.25 (2.16, 2.32)	-2.95	**0.003**
Phosphorus (mmol/L), M (Q_1_, Q_3_)	1.25 (1.06, 1.40)	1.25 (1.07, 1.40)	1.25 (1.06, 1.39)	-0.30	0.760
Urea (mmol/L), M (Q_1_, Q_3_)	17.6 (8.7, 29.2)	15.4 (8.3, 27.5)	20.8 (16.2, 35.6)	-3.98	**<0.01**
UA (umol/L), M (Q_1_, Q_3_)	360.0 (292.0, 430.0)	347.0 (270.0, 409.0)	406.5 (339.3, 492.8)	-6.01	**<0.01**
Vitamin D (ng/mL), M (Q_1_, Q_3_)	20.4 (14.7, 28.2)	19.1 (14.7, 27.9)	22.5 (14.1, 31.0)	-1.53	0.126
iPTH (pg/mL), M (Q_1_, Q_3_)	50.0 (34.2, 78.0)	49.0 (34.0, 74.0)	53.5 (38.3, 104.5)	-1.74	0.083

Z, Mann-Whitney test; χ², Chi-square test; M, Median; Q₁, 1st Quartile; Q₃, 3st Quartile. LVH, Left ventricular hypertrophy; RAAS, Renin-angiotensin-aldosterone system; SGLT2i, Sodium-glucose transporter 2 inhibitors; WBC, White blood cell; RBC, Red blood cell; Net, Neutrophil; Lym, Lymphocyte; Mono, Monocyte; NLR, Neutrophil-to-lymphocyte ratio; MLR, Monocyte-to-lymphocyte ratio; PLR, Platelet-to-lymphocyte ratio; SII, Systemic immune inflammation index; UA, Uric acid; iPTH, Intact parathyroid hormone. Bold values indicate *P*<0.05.

### 4.3 Logistic regression analysis of LVH in the participants

Univariate logistic regression analysis indicated that gender, neutrophil, SII, calcium, UA, and urea were associated with LVH (all *P<*0.05). Multivariate logistic regression analysis revealed that neutrophil (*OR* = 1.31, 95%*CI*: 1.06 ~ 1.62, *P* = 0.011), SII (*OR* = 1.01, 95%*CI*: 1.01 ~ 1.01, *P* = 0.011), UA (*OR* = 1.01, 95%*CI*: 1.01 ~ 1.01, *P<*0.01), and urea (*OR* = 1.02, 95%*CI*: 1.01 ~ 1.03, *P* = 0.002) are positively associated with LVH. In contrast, male gender (*OR* = 0.33, 95%*CI*: 0.20 ~ 0.56, *P<*0.01) and calcium (*OR* = 0.07, 95%*CI*: 0.01 ~ 0.34, *P* = 0.001) are negatively associated with LVH, as shown in the **Table 3**. The stage of DKD did not significantly affect neutrophil (interaction *P* = 0.09) or SII (interaction *P* = 0.22). The collinearity test tolerances were all>0.75, and the variance inflation factors (VIF) were all<1.35, suggesting no collinearity among the independent variables.

**Table 3:** Logistic regression analysis of LVH in the participants.

Variables	Single factor					Multiple factors				
	β	S.E	Wald	*P*	*OR* (95%*CI*)	β	S.E	Wald	*P*	*OR* (95%*CI*)
Gender										
Female					1.00					
Male	-0.79	0.23	-3.47	**<0.01**	0.45 (0.29 ~ 0.71)	-1.10	0.27	-4.09	**<0.01**	0.33 (0.20 ~ 0.56)
Ethnicity										
Non-han ethnicity					1.00					
Han ethnicity	0.12	0.36	0.32	0.751	1.12 (0.55 ~ 2.29)					
Hypertention										
Without hypertention					1.00					
With hypertention	0.15	0.27	0.55	0.585	1.16 (0.68 ~ 1.99)					
CVD										
Without CVD					1.00					
With CVD	0.11	0.23	0.48	0.634	1.12 (0.71 ~ 1.76)					
DKD stage										
G2					1.00					
G3a	-0.44	0.30	-1.47	0.141	0.64 (0.36 ~ 1.16)					
G3b	-0.39	0.31	-1.25	0.211	0.68 (0.37 ~ 1.25)					
G4	0.33	0.33	1.00	0.318	1.38 (0.73 ~ 2.62)					
Age	-0.00	0.01	-0.23	0.818	1.00 (0.98 ~ 1.02)					
Net (×10^9^/L)	0.33	0.08	3.97	**<0.01**	1.39 (1.18 ~ 1.64)	0.27	0.11	2.54	**0.011**	1.31 (1.06 ~ 1.62)
NLR	0.08	0.07	1.17	0.242	1.09 (0.94 ~ 1.25)					
PLR	0.00	0.00	1.52	0.128	1.00 (1.00 ~ 1.01)					
SII (×10^9^/L)	0.01	0.00	4.05	**<0.01**	1.01 (1.01 ~ 1.01)	0.01	0.00	2.56	**0.011**	1.01 (1.01 ~ 1.01)
Calcium (mmol/L)	-1.94	0.73	-2.67	**0.008**	0.14 (0.03 ~ 0.60)	-2.69	0.83	-3.26	**0.001**	0.07 (0.01 ~ 0.34)
UA (umol/L)	0.01	0.00	5.82	**<0.01**	1.01 (1.01 ~ 1.01)	0.01	0.00	6.13	**<0.01**	1.01 (1.01 ~ 1.01)
Urea (mmol/L)	0.02	0.01	3.11	**0.002**	1.02 (1.01 ~ 1.03)	0.02	0.01	3.07	**0.002**	1.02 (1.01 ~ 1.03)

OR, Odds Ratio; CVD, Cardiovascular Disease; Net, Neutrophil; PLR, Platelet-to-lymphocyte Ratio; SII, Systemic Immune Inflammation Index; UA, Uric Acid. Bold values indicate *P*<0.05.

### 4.4 ROC curve of the predictive assessment of LVH in patients with DKD based on basic variables combined with routine blood count-derived inflammatory markers

**Figure 1:**
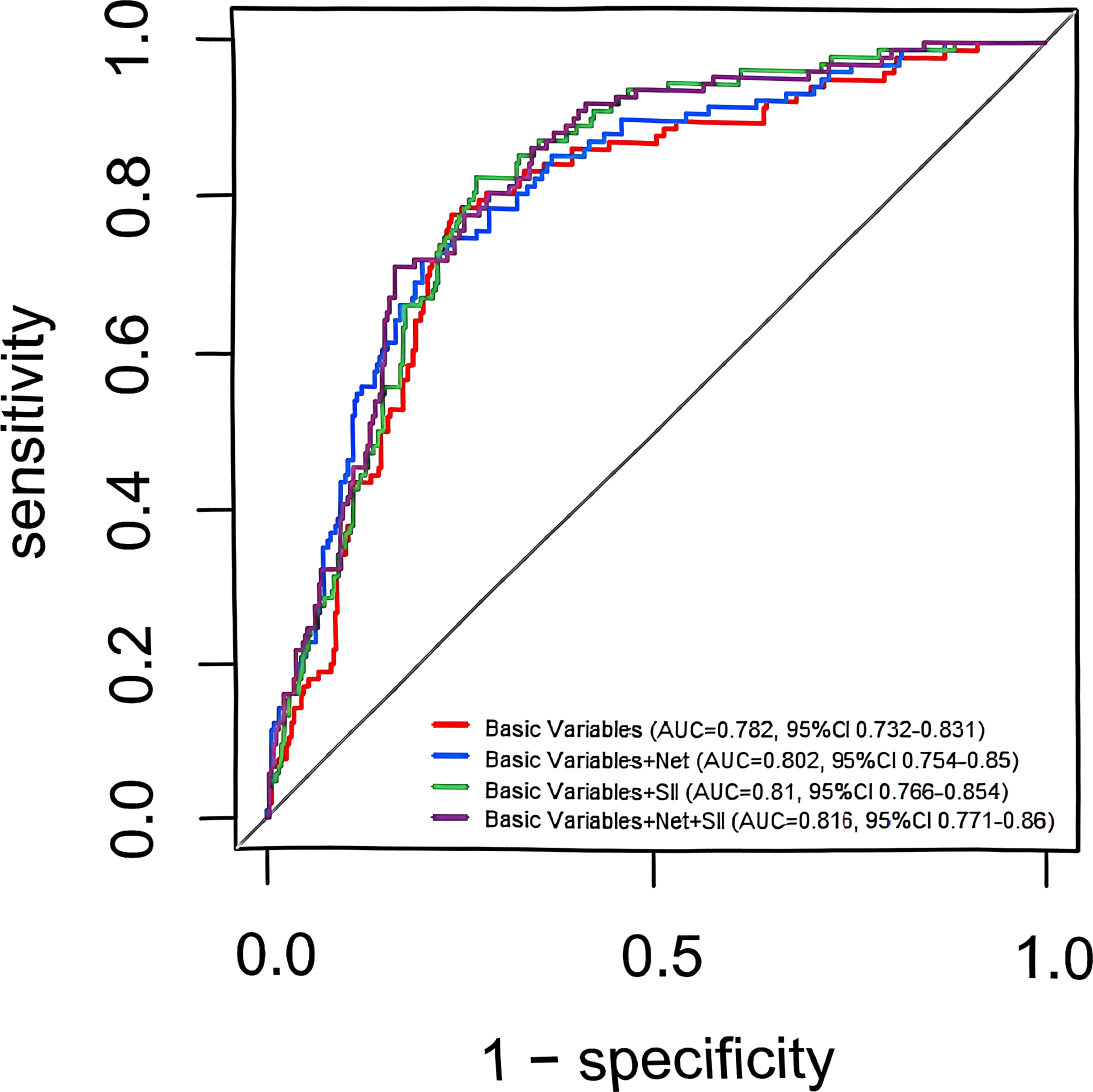
ROC curve of LVH in patients with DKD based on basic variables combined with routine blood count-derived inflammatory markers.

The AUC for the combination of basic variables (gender, calcium, UA, urea) with neutrophil and SII was 0.816 (95%*CI*: 0.771 ~ 0.860), while the AUC for the combination of basic variables with SII was 0.810 (95%*CI*: 0.766 ~ 0.854). Both of these values exceeded the AUC for the basic variables alone, which was 0.782 (95%*CI*: 0.732 ~ 0.831), with significant differences observed (all *P* < 0.05). The AUC for the combination of basic variables with neutrophil was 0.802 (95%*CI*: 0.754 ~ 0.850), which was greater than the AUC for the basic variables alone (0.782, 95%*CI*: 0.732 ~ 0.831), although this difference was not statistically significant (*P*>0.05). The IDI for the aforementioned models were 0.06, 0.04, and 0.05, respectively, as illustrated in **Figure 1** and **Table 4**.

**Table 4:** The AUC (95%CI), IDI and P values of the four models compared with basic model.

Models	AUC	*P*	95%*CI*	IDI
Basic Variables	0.782		0.732 ~ 0.831	
Basic Variables+Net	0.802	0.10	0.754 ~ 0.850	0.05
Basic Variables+SII	0.810	0.02	0.766 ~ 0.854	0.04
Basic Variables+Net+SII	0.816	0.01	0.771 ~ 0.860	0.06

Net, Neutrophil; SII, Systemic Immune Inflammation Index; AUC, Area Under the Curve; CI, Confidence Interval; IDI, Integrated Discrimination Improvement.

### 4.5 The optimal cut-off value of neutrophil and SII

The results of the analysis to determine the optimal cut-off values of neutrophil and SII for predicting LVH in patients with DKD, utilizing the Youden index maximization method, indicate the following: the optimal cut-off value for neutrophil is 4.14×10^9^/L, with a corresponding sensitivity of 72.6% and specificity of 52.4%, yielding a Youden index of 0.250. In contrast, the optimal cutoff value for SII is 491.78×10^9^/L, which demonstrates a sensitivity of 84.9% and a specificity of 57.2%, resulting in a Youden index of 0.421. Detailed information can be found in **Table 5**.

**Table 5:** The optimal cut-off value of neutrophil and SII.

Variables	Optimal cut-off value (×10^9^/L)	Sensitivity (%)	Specificity (%)	Youden Index
Net	4.135	72.6	52.4	0.250
SII	491.78	84.9	57.2	0.421

Net, Neutrophil; SII, Systemic immune inflammation index.

## 5 Discussion

In the cross-sectional study involving 419 participants, we found that LVH in individuals with DKD is influenced by both metabolic disorders and immune inflammation. Multivariate logistic regression analysis revealed that, in addition to traditional metabolic indicators such as UA and urea, neutrophil and SII serve as independent positive correlation factors for LVH. Conversely, male gender and calcium are identified as negative correlation factors. The stage of DKD did not significantly affect neutrophil or SII. Importantly, collinearity testing (VIF<1.35) confirmed the independent contributions of SII and neutrophils in the model. In terms of predictive performance, the inclusion of SII, neutrophil, and SII to the basic model-comprising gender, calcium, uric acid, and urea-resulted in a statistically significant increase in the AUC. Additionally, the IDI demonstrated significant positive benefits. These findings indicate that the integration of inflammatory indicators, such as SII, alongside traditional clinical assessments, can substantially enhance the accuracy of identifying the risk of cardiac remodeling in patients with DKD.

The principal finding of this study is the independent correlation between the SII, neutrophil, and LVH, which aligns with the findings of Wang L et al. and robustly supports the “immune-inflammation pathogenic hypothesis” (18). Patients with DKD frequently exist in a state of persistent low-grade systemic inflammation. Neutrophils serve as the primary defense mechanism of innate immunity. Elevated neutrophil not only indicate the body’s stress response but also play a direct role in cardiovascular damage. Activated neutrophils can induce vascular endothelial dysfunction and facilitate the transformation of fibroblasts into myofibroblasts through the release of reactive oxygen species, myeloperoxidase, and neutrophil extracellular traps, thereby contributing to myocardial interstitial fibrosis and hypertrophy (19). SII, a composite indicator that combines neutrophil, lymphocyte, and platelet, more effectively reflects the balance between pro-inflammatory and anti-inflammatory states in the body than any single indicator (20). In this study, while NLR and PLR demonstrated a certain association in univariate analysis, multivariate regression revealed that neutrophil (*OR* = 1.31) and SII (*OR* = 1.01) were more robust, indicating its greater clinical relevance in assessing LVH.

This study reaffirms that UA and urea serve as independent positive correlation factors for LVH in patients with DKD. Hyperuricemia is prevalent among individuals with DKD, and Elisa G et al. demonstrated through a meta-analysis that hyperuricemia may predict an increase in left ventricular mass fraction in women (21). Additionally, the URRAH study conducted by Maria L et al. established that UA are independently associated with left ventricular mass index. Furthermore, the co-occurrence of hyperuricemia and LVH emerges as a robust independent predictor of cardiovascular mortality in both men and women (22). Hyperuricemia can lead to endothelial dysfunction by inhibiting nitric oxide synthase and activating the local RAAS, which stimulates cardiomyocyte hypertrophy. As a representative of uremic toxins, increased urea levels are frequently associated with enhanced protein formylation modifications, resulting in mitochondrial dysfunction and cardiomyocyte apoptosis. The findings of this study underscore the importance of actively addressing hyperuricemia and azotemia, alongside managing blood sugar and blood pressure, to achieve cardioprotection in the treatment of DKD (23).

This study identified male gender as a negative correlation factor for LVH (*OR* = 0.33), indicating that female patients with DKD face a significantly higher risk of LVH compared to their male counterparts. This finding aligns with the PURSUIT-HFpEF study conducted by Yohei S et al. (24), which demonstrated that elderly female patients with heart failure and preserved ejection fraction are more prone to developing diastolic dysfunction and experiencing poorer clinical outcomes, with LVH and stiffness serving as the underlying pathological mechanisms (25). However, previous studies (26) have indicated that men typically experience faster disease progression and a higher risk of cardiovascular events in patients with DKD. The gender trend observed in this study is contrary to these findings and may be influenced by the specific characteristics of the study participants, including inclusion criteria, disease progression, and the distribution of comorbidities. Current ultrasonic diagnostic criteria for LVH reveal gender differences; the left ventricular mass index (LVMI) threshold for women (≥95 g/m²) is lower than that for men (≥115 g/m²). This discrepancy may lead to a higher likelihood of women being classified as having LVH, thereby potentially exaggerating the “real” risk of LVH in women.

Additionally, calcium was identified as a negative correlation factor (*OR* = 0.07) in multivariate analysis, indicating that hypocalcemia serves as a risk factor for LVH. This finding underscores the significant role of chronic kidney disease-mineral and bone disorders (CKD-MBD) in cardiac remodeling. In stages 2–4 of DKD, impaired activation of vitD results in decreased intestinal calcium absorption, leading to hypocalcemia, which subsequently triggers a secondary increase in PTH levels. Prolonged elevation of PTH not only contributes to bone loss but also acts as a “uremic toxin” that directly interacts with PTH receptors on cardiomyocytes, resulting in intracellular calcium overload and the activation of hypertrophy signaling pathways (27). Therefore, maintaining serum calcium levels within the normal range is essential not only for bone health but also for preventing secondary hyperparathyroidism (SHPT)-mediated myocardial damage.

This study demonstrated that the integration of SII with basic variables-specifically gender, calcium, UA, and Urea-resulted in an AUC of 0.810 for predicting DKD patients with LVH. This AUC was significantly higher than that of the basic model, which had an AUC of 0.782 (*P* = 0.024). When the basic model was further enhanced by incorporating both neutrophil and SII, the AUC increased to 0.816 (*P* = 0.013). Although the addition of neutrophil alone raised the AUC to 0.802, this increase did not achieve statistical significance (*P* = 0.10). The IDI values for the basic model combined with neutrophil, SII, and both indicators were 0.05, 0.04, and 0.06, respectively. These findings indicate that the inclusion of inflammatory markers has improved the model’s ability to reclassify risk. The results indicate that the SII, which integrates information from platelets, neutrophils, and lymphocytes, enhances the predictive capability for LVH in patients with DKD more reliably than the neutrophil alone. This finding aligns with previous studies that have examined SII in the context of CVD. For instance, SII has been linked to left ventricular remodeling in conditions such as coronary artery disease, heart failure, and hypertension. This study further expands the predictive significance of SII within the DKD patients, demonstrating that SII continues to provide independent prognostic information even after adjusting for traditional risk factors, including calcium, UA, and urea.

It is important to note that while neutrophil was independently associated with LVH in multivariate regression analysis (*OR* = 1.31, *P* = 0.011), its contribution to model discrimination did not achieve statistical significance. The SII, as a composite measure, may more effectively capture the overall activation status of the immune-inflammatory network. Furthermore, the optimal cut-off value for SII is 491.78×10^9^/L, demonstrating a sensitivity of 84.9%. This suggests that SII may better mitigate the rate of missed diagnoses when screening for DKD in patients with LVH, making it suitable for preliminary clinical risk assessment. Conversely, the optimal cut-off value for neutrophil is 4.14×10^9^/L, which exhibits low sensitivity (72.6%) and low specificity (52.4%). This indicates a high rate of misjudgment when neutrophil is used in isolation as a diagnostic marker, suggesting that it is more appropriately utilized in conjunction with other indicators.

The findings of this study hold significant clinical translational implications. In routine clinical practice, particularly in primary care settings or regions with limited medical resources, the implementation of routine echocardiography or cardiac magnetic resonance imaging screening for all patients with DKD is often constrained by cost and equipment availability. Furthermore, traditional heart failure biomarkers, such as NT-pro-BNP, are relatively expensive to measure. In contrast, complete blood count is one of the most fundamental, cost-effective, and widely utilized routine tests in outpatient settings. This study illustrates that incorporating low-cost routine parameters into the SII offers clinicians a straightforward, non-invasive, and economical risk assessment tool. It highlights that in everyday diagnosis and treatment, physicians can utilize routine blood reports to swiftly calculate the SII, enabling the identification of DKD patients at elevated risk for LVH, thereby optimizing clinical disease management strategies for this high-risk population.

This study has several limitations. First, as a cross-sectional investigation, it cannot establish a causal relationship between SII and the occurrence of LVH, nor can it assess the long-term effects of dynamic changes in SII on cardiac outcomes. Second, the sample for this study was derived from a single center and predominantly consisted of Han Chinese participants, which may restrict the generalizability of the findings to other ethnic groups or regions. Third, this study did not include a prior sample size calculation, and only 23 patients (21.7%) with DKD stage G4 exhibited LVH. The number of patients at this stage was relatively limited. Forth, although we adjusted for various covariates in the multifactorial model, we cannot entirely exclude the influence of unmeasured potential residual confounding factors, such as patients’ dietary habits and genetic polymorphisms, on cardiac remodeling. Furthermore, the impact of drug use duration or adherence on LVH cannot be discounted. Fifth, regarding predictive performance, while the improvement in AUC of the joint model relative to the basic model is statistically significant, the IDI also indicates favorable outcomes. But in the future, it will be essential to incorporate more advanced biomarkers and imaging techniques, such as NT-pro-BNP or cardiac magnetic resonance, to further validate the application value of SII in cardiovascular risk stratification for DKD within a multi-center, large-sample prospective cohort.

## 6 Conclusion

In summary, among patients with DKD G2 ~ G4, the routine blood count-derived inflammatory marker SII and peripheral blood neutrophil serve as independent positive correlation factors for LVH. The AUC of the basic variables, when combined with SII and neutrophil, as well as SII alone, demonstrates a statistically significant improvement over the AUC of the basic model. Additionally, IDI analysis corroborated that these combinations provide incremental value in enhancing risk prediction. Additionally, female gender, hypocalcemia, hyperuricemia, and high urea are important indicators for identifying high-risk patients. In routine diagnosis and treatment, clinicians should prioritize the readily obtainable complete blood count and calculate SII to assist in identifying the risk of early cardiac damage, thus facilitating the early diagnosis and treatment of cardiorenal complications associated with DKD.

## Data Availability

The raw data supporting the conclusions of this article will be made available by the authors, without undue reservation.
